# Case Report: A case of severe hypotension induced by nimotuzumab in a nasopharyngeal carcinoma patient

**DOI:** 10.3389/fimmu.2026.1807371

**Published:** 2026-03-18

**Authors:** Wenhui Liu, Ping Qin, Feiyan Xiao, Bao Sun

**Affiliations:** 1 Department of Pharmacy, The Second Xiangya Hospital, Central South University, Changsha, China; 2 Department of Pharmacy, Guilin People’s Hospital, Guilin, China; 3 Center for Clinical Trial and Research, The Second Xiangya Hospital, Central South University, Changsha, China

**Keywords:** adverse drug reaction, EGFR monoclonal antibody, hypotension, nasopharyngeal carcinoma, nimotuzumab

## Abstract

Nimotuzumab, a humanized immunoglobulin G1 monoclonal antibody, is mainly used in combination with radiotherapy for the treatment of stage III/IV nasopharyngeal carcinoma (NPC) or locally advanced head and neck squamous-cell carcinoma (HNSCC) with positive epidermal growth factor receptor (EGFR) expression. Although the drug has a favorable safety profile, it is associated with various adverse reactions, among which hypotension is relatively uncommon. Clinically, hypotension caused by nimotuzumab mainly manifests as mild blood pressure (BP) reduction, which can be relieved via rest. This case reports a 65-year-old male patient with advanced NPC who developed severe persistent hypotension after receiving radiotherapy combined with 5 cycles of nimotuzumab treatment. After excluding related factors such as cardiac function, endocrine function, thyroid function, inflammatory factors and other drugs, the adverse reaction was considered to be closely associated with nimotuzumab. After discontinuing the drug and giving continuous norepinephrine to increase BP, the patient’s BP returned to stable. This case suggests that although nimotuzumab-related hypotension is mostly mild and reversible, BP monitoring should still be strengthened to maintain vigilance against severe hypotension and intervene promptly in clinical practice.

## 1 Introduction

NPC is a malignant tumor with distinct geographical distribution, which has a high incidence rate in southern China and Southeast Asia (1). Studies have shown that nearly 85% of NPC patients exhibit high EGFR expression, and this indicator is closely related to poor prognosis (2). Nimotuzumab is a humanized monoclonal antibody targeting EGFR, which can specifically block the EGFR signaling pathway and enhance the sensitivity of tumor cells to radiotherapy. Due to its high selectivity, nimotuzumab exhibits a good safety profile (3). Hypotension is a rare adverse reaction of nimotuzumab, and phase II clinical trial data showed that its incidence rate is 2.86%, while post-marketing clinical reports in China indicate that hypotension related to nimotuzumab is rare (≥1/10,000, <1/1,000) or very rare (<1/10,000) (4). This article reports a case of severe hypotension in an NPC patient during the treatment of nimotuzumab, and the patient’s BP was effectively controlled after active treatment. To our knowledge, this is the first domestic case report on severe hypotension caused by nimotuzumab, which can provide important references for the clinical safety of this drug.

## 2 Case presentation

A 65-year-old male patient who had no previous history of hypotension and denied family genetic history was admitted in August 2025 due to recurrent minor epistaxis. After admission, he was diagnosed with NPC (TNM stage III) through examinations. Immunohistochemistry indicated that EGFR was positivite. The patient initially received 3 cycles of chemotherapy (cisplatin 40 mg, d1-3; docetaxel 110 mg, d1) combined with immunotherapy (tislelizumab 200 mg, d1). Evaluation by nasal and neck MRI showed a partial response. To further pursue curative treatment, the patient received concurrent radiotherapy and 4 cycles of targeted therapy (nimotuzumab 200 mg, q1w) from November 11 to December 1, 2025. During the treatment period, the patient reported slight pain when swallowing but no other specific discomfort. The patient’s mental status, diet, sleep, bowel movements, and urination were normal, and there was no significant change in body weight. On December 10, the patient’s BP suddenly dropped to 74/56 mmHg; after administration of hydrocortisone (200 mg/d), the BP rose to 109/69 mmHg. On December 11, the patient continued to receive concurrent radiotherapy and targeted therapy according to the original plan. On December 14, the patient’s BP further dropped to 59/42 mmHg, with a heart rate of 72 beats per minute, respiratory rate of 20 breaths per minute, pulse oxygen saturation of 96%, and accompanied with dizziness. Hydrocortisone was immediately discontinued, and a norepinephrine group (15 mg norepinephrine + 50 ml 5% glucose injection) was given via continuous infusion pump (25 mg/d). The dosage was dynamically adjusted, BP, oxygen saturation, and electrocardiographic conditions were continuously monitored.

The patient denied discomfort such as chest tightness, chest pain, palpitations, shortness of breath, or paroxysmal nocturnal dyspnea. Auxiliary examination results showed age-appropriate cortical atrophy, no obvious abnormalities in cardiac function, unobstructed blood flow, which basically ruled out the possibility of cardiogenic hypotension. Laboratory test results: adrenocorticotropic hormone (ACTH) 23 ng/L (reference range: 0~46 ng/L) and cortisol 720.4 nmol/L (reference range: 145.4~619.4 nmol/L) at 8 A.M.; ACTH 8.6 ng/L (reference range: 1.6~16.6 ng/L) and cortisol 452.7 nmol/L (reference range: 94.9~462.4 nmol/L) at 4 P.M. Two days after the patient stopped using hydrocortisone, the rechecked cortisol and ACTH indicators did not decrease, and hormone therapy had no significant effect on improving BP, which could exclude hypotension associated with cortisol insufficiency. Free triiodothyronine (FT3) 5.5 pmol/L (reference range: 3.5~6.5 pmol/L); free thyroxine (FT4) 12.7 pmol/L (reference range: 11.5~22.7 pmol/L); thyroid-stimulating hormone (TSH) 2.05 μIU/mL (reference range: 0.55~4.78 μIU/mL), thyroid function test results were normal, which essentially ruled out hypotension caused by thyroid insufficiency. C-reactive protein (CRP) 53.08 mg/L (reference range: 0~6 mg/L); erythrocyte sedimentation rate (ESR) 53 mm/h (reference range: 0~15 mm/h); procalcitonin (PCT) 0.133 ng/mL (reference range: 0.00~0.05 ng/mL); interleukin-6 (IL-6) 29.10 pg/mL (reference range: 0.00~7.00 pg/mL), the patient’s inflammatory markers were not significantly elevated, and there was no accompanying fever, which basically ruled out hypotension caused by infection or cytokine release syndrome. The blood pressure of the patient was measured using an ambulatory blood pressure monitor. During the measurement, the patient was in a supine position, the cuff was properly fitted to the right upper arm and at the same level as the heart, which ruled out orthostatic hypotension.

**Figure 1:**
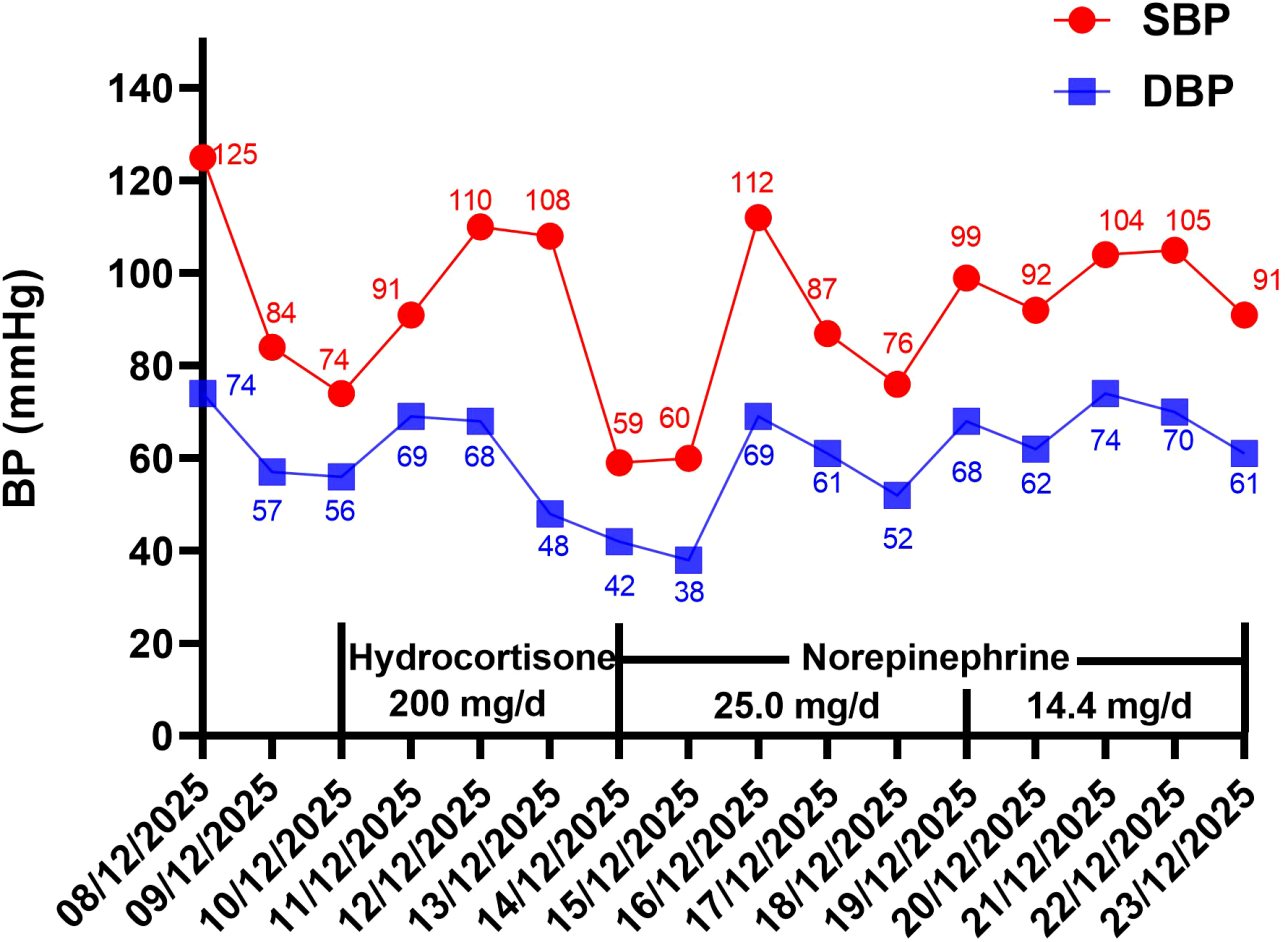
The BP changes of the patient during hospitalization.

Continuous norepinephrine infusion was maintained to support BP. From December 14 to December 18, the patient’s BP fluctuated greatly, with systolic blood pressure (SBP) maintained at 55~115 mmHg and diastolic blood pressure (DBP) maintained at 40~70 mmHg. The patient’s BP gradually stabilized from December 19 to December 23, with SBP maintained at 90~105 mmHg and DBP maintained at 60~75 mmHg. Therefore, the dosage of the norepinephrine was adjusted to 14.4 mg/d. The patient’s BP and vital sign indicators were shown in **Figure 1** and **Table 1** during hospitalization. The patient was discharged after completing radiotherapy on December 23. At discharge, the patient was advised to continue monitoring BP. We conducted a telephone follow-up with the patient. Since discharge, the patient has not used the nimotuzumab or vasopressor medications again, and currently his blood pressure is well-controlled without any signs of hypotension through telephone follow-up.

**Table 1:** The HR, RR, O_2_ sat and temperature of patient during hospitalization.

Timeline	12-08	12-9	12-10	12-11	12-12	12-13	12-14	12-15	12-16	12-17	12-18	12-19	12-20	12-21	12-22	12-23
HR (beats per minute)	96	67	87	75	70	66	72	80	78	72	78	86	80	92	75	78
RR (acts per minute)	20	18	19	20	20	20	20	20	22	20	20	20	20	20	20	20
O_2_ sat (%)	98	95	96	95	98	99	96	98	98	97	96	96	95	95	95	98
Temperature (°C)	37	36.5	36.8	36.2	36.6	36.5	36	36.8	36.5	36.8	36.5	36.8	36.4	36.3	36.9	36.5

RR, respiratory rate; HR, heart rate; BP, blood pressure; O_2_ sat, oxygen saturation.

## 3 Discussion

In this case, an NPC patient developed severe hypotension after nimotuzumab treatment. According to the *Common Terminology Criteria for Adverse Events* (CTCAE Version 5.0) (5), this adverse reaction was graded as Grade 3, which is classified as severe hypotension. The patient’s hypotensive symptoms were effectively improved after continuous infusion treatment with norepinephrine.

After the patient developed hypotension, we performed a series of examinations, including cardiac color doppler ultrasound, ACTH, cortisol, thyroid function indicators and inflammatory markers, to rule out hypotension caused by cardiac issues, cortisol insufficiency, thyroid dysfunction and infection. The patient’s hypotension symptoms occurred during nimotuzumab targeted therapy, and the BP showed a progressive downward trend as the treatment cycle progressed. According to the Naranjo Adverse Reaction Assessment Scale (6), nimotuzumab scored 6 points, suggesting a “probable” association between the drug and the hypotension adverse reaction (**Table 2**). Although docetaxel (7, 8), cisplatin (8), and tislelizumab (9) have potential risks of causing hypotension, such adverse reactions mostly occur during medication. However, when the patient developed hypotension symptoms, chemotherapy and immunotherapy drugs had been discontinued for 2 months. Therefore, it was considered that the hypotension of this patient was more strongly associated with nimotuzumab.

**Table 2:** Naranjo's ADR evaluation/probability scale.

Question	Score of the question			Suspected drug			
	Yes	No	Don't know	Docetaxel	Cisplatin	Tislelizumab	Nimotuzumab
Are there previous conclusion reports on this reaction?	+1	0	0	+1	+1	+1	+1
Did the adverse event appear after the suspect drug was administered?	+2	-1	0	+2	+2	+2	+2
Did the adverse reaction improve when the drug was discontinued or a specific antagonist was administered?	+1	0	0	0	0	0	+1
Did the adverse reaction reappear when drug was readministered?	+2	-1	0	0	0	0	+2
Are there alternate causes [other than the drug] that could solely have caused the reaction?	-1	+2	0	-1	-1	-1	-1
Did the reaction reappear when a placebo was given?	-1	+1	0	0	0	0	0
Was the drug detected in the blood [or other fluids] in a concentration known to be toxic?	+1	0	0	0	0	0	0
Was the reaction more severe when the dose was increased or less severe when the dose was decreased?	+1	0	0	0	0	0	0
Did the patient have a similar reaction to the same or similar drugs in any previous exposure?	+1	0	0	0	0	0	0
Was the adverse event confirmed by objective evidence?	+1	0	0	0	0	0	+1
Causality Score				2	2	2	6

The Naranjo scale assigns a causality score, which is the sum of the scores of all questions, that falls into one of four causality types: *doubtful* (≤ 0),* possible *(1 − 4),* probable *(5 − 8), and *definite* (≥ 9).

Nimotuzumab is a humanized immunoglobulin G1 monoclonal antibody (10) with moderate affinity for EGFR. It can selectively bind to tumor cells with high EGFR expression, resulting in high drug uptake in tumor tissues and low uptake in normal tissues, thereby reducing the incidence of adverse reactions (11). Common adverse reactions of the drug mainly include skin reactions, gastrointestinal reactions, and hematopoietic system reactions, while the incidence of hypotension is low. To our knowledge, this case is the first case report on severe hypotension caused by nimotuzumab.

Other EGFR monoclonal antibodies, such as cetuximab and panitumumab, have been reported to cause severe hypotension (12, 13). However, the severe hypotension is caused by an allergic shock resulting from an infusion reaction, with clinical manifestations including stridor/bronchospasm, angioedema (especially laryngeal edema), hypotension, pruritus, urticaria, gastrointestinal sequelae (nausea, vomiting, pain, diarrhea) and cardiac-related issues (arrhythmias). Most of these reactions occurred within minutes of the first infusion (14). Regarding this case, the patient’s hypotension occurred on day 3 after the 5th cycle of nimotuzumab treatment, with no symptoms of infusion reaction. These findings suggest that this case is different from the previously reported cases of hypotension caused by EGFR monoclonal antibodies, and there may be other mechanisms that cause a decrease in blood pressure. Current research proposed that EGFR is involved in the regulation of vasoconstrictive function in vascular smooth muscle cells (VSMCs). Relevant literature pointed out (15) that the SBP, DBP, and mean arterial pressure (MAP) were all significantly reduced in mice with specific knockout of EGFR in vascular smooth muscle cells. Nimotuzumab may inhibit EGFR in vascular smooth muscle cells, leading to decreased vasoconstrictive capacity and reduced peripheral resistance, thereby causing hypotension. Further studies indicate that G protein-coupled receptor (GPCR) agonists could promote EGFR signal transduction, a process known as EGFR “transactivation”. Angiotensin II (Ang II) is a GPCR agonist that plays an important role in the development and progression of various cardiovascular diseases, including hypertension and atherosclerosis, through the “transactivation” of EGFR (16, 17). Therefore, EGFR inhibition may antagonize the vasoconstrictive effect of Ang II by blocking EGFR “transactivation”, leading to vasodilation and reduced vascular resistance, which provides further evidence for the role of nimotuzumab in blood pressure regulation by modulating vascular tone. In addition, nimotuzumab can also reduce the overall microvessel density (MVD) and exert significant anti-angiogenic effects (18, 19), leading to vascular endothelial cell apoptosis, increasing vascular permeability, reducing effective circulating blood volume, and further aggravating the hypotension symptoms.

Previous cases reported that hypotension induced by EGFR inhibitors has generally been treated with epinephrine (20) or ephedrine (12). However, pressor drugs such as epinephrine and ephedrine had a strong agonistic effect on β-receptors, which might produce other side effects. This patient only presented with decreased blood pressure accompanied by mild dizziness, with normal heart rate, respiratory rate, and blood oxygen saturation. To avoid other side effects caused by epinephrine or ephedrine, we chose norepinephrine, which has a weak agonistic effect on β-receptors, and successfully controlled the patient’s blood pressure.

However, this report has certain limitations. Firstly, this is a single-center, single-case report, lacking support from large-sample data, which limits the extrapolation of the research conclusions; secondly, the exploration of the mechanism of hypotension in this case lacks clear direct evidence, and further research is still needed in the future to deeply explore the specific mechanism of nimotuzumab-induced hypotension.

## 4 Conclusion

This case suggests that nimotuzumab treatment may cause severe hypotension in patients. During the clinical application of this drug, it is essential to closely monitor the changes in patients’ BP.

## Data Availability

The original contributions presented in the study are included in the article/supplementary material. Further inquiries can be directed to the corresponding authors.
