# Correction: Case Report: A case of severe hypotension induced by nimotuzumab in a nasopharyngeal carcinoma patient

**DOI:** 10.3389/fimmu.2026.1875677

**Published:** 2026-05-18

**Authors:** Wenhui Liu, Ping Qin, Feiyan Xiao, Bao Sun

**Affiliations:** 1 Department of Pharmacy, The Second Xiangya Hospital, Central South University, Changsha, China; 2 Department of Pharmacy, Guilin People’s Hospital, Guilin, China; 3 Center for Clinical Trial and Research, The Second Xiangya Hospital, Central South University, Changsha, China

**Keywords:** adverse drug reaction, EGFR monoclonal antibody, hypotension, nasopharyngeal carcinoma, nimotuzumab

In the published article, an author name was incorrectly written as Feiyao Xiao^3*^. The correct spelling is Feiyan Xiao^3*^.

The authors apologize for this error and state that this does not change the scientific conclusions of the article in any way.

The original article has been updated.

